# Evaluation of the role development and implementation of advanced practice nurses in an Austrian university hospital: a mixed methods research protocol

**DOI:** 10.1136/bmjopen-2026-119182

**Published:** 2026-07-31

**Authors:** Kathrin Julia Pann, Simone Elisabeth Gruber, Roland Eßl-Maurer, Franziska Moser, Andre Ewers

**Affiliations:** 1 Nursing Management, Coordination of Clinical Nursing Science and Research, University Hospital Salzburg, Paracelsus Medical University, Salzburg, Austria; 2 Doctoral Program Nursing and Allied Health Sciences, Department of Nursing Science and Practice, Paracelsus Medical University, Salzburg, Austria; 3 Nursing Management, University Hospital Salzburg, Paracelsus Medical University, Salzburg, Austria; 4 Nursing Management, Coordination Development Clinical Nursing Practice, University Hospital Salzburg, Paracelsus Medical University, Salzburg, Austria; 5 Nursing Management, Director of Nursing, University Hospital Salzburg, Paracelsus Medical University, Salzburg, Austria

**Keywords:** Nursing research, Nurses, Hospitals, Surveys and Questionnaires, QUALITATIVE RESEARCH

## Abstract

**Abstract:**

**Introduction:**

Advanced practice nurses play a vital role in addressing the growing complexity of healthcare. Although well-established internationally, advanced practice nursing in Austria remains in its early stages. The University Hospital Salzburg introduced its first advanced practice nurses in 2017, and in 2020 launched a comprehensive initiative to develop and implement advanced practice nursing roles across multiple specialties. This initiative included role development based on international frameworks, the creation of an advanced practice nurse network, scientific mentoring and an adapted skill and grade mix. This study aims to evaluate the development and implementation of advanced practice nursing roles at the University Hospital Salzburg across multiple disciplines and at the system level. It will analyse their impact on clinical, professional and organisational outcomes, integrating the perspectives of various healthcare professionals as well as the advanced practice nurses.

**Methods and analysis:**

This mixed methods study will follow an explanatory sequential design. Advanced practice nurses, registered nurses, nurse assistants, nurse managers and physicians will be invited to participate. The quantitative phase will employ validated instruments and research team-developed questionnaires. Quantitative results will inform the development of the interview guide and sampling strategy for the qualitative phase. In the qualitative phase, expert interviews and focus groups will be conducted, with data analysed thematically. Quantitative and qualitative findings will be integrated using a joint display across clinical, professional and organisational levels. The study will be conducted from June 2025 to November 2027 at an Austrian university hospital.

**Ethics and dissemination:**

According to the Ethics Committee of Salzburg, formal ethical approval was not required. Study findings will be disseminated through publications in peer-reviewed journals, presentations at scientific conferences and internal events for participants.

**Estimated start of the qualitative phase:**

July 2026.

**Trial registration number:**

DRKS00036497.

**STRENGTHS AND LIMITATIONS OF THIS STUDY**

- This study evaluates the impact of advanced practice nurse role development and implementation from a system-level perspective.
- It underscores the importance of integrating quantitative and qualitative data to address the complexity of evaluating advanced practice nurse roles.
- This study protocol may serve as a resource for countries seeking to evaluate advanced practice nursing roles during early stages of development.
- While several validated instruments are employed, the study also includes a self-developed questionnaire for which formal psychometric validation is still pending.

## Introduction

 The demographic shift and the growing prevalence of chronic diseases are increasing the complexity of healthcare and nursing. At the same time, advances in technology, therapeutic techniques and diagnostic procedures require the modernisation and adaptation of care models and healthcare delivery structures.^1^ On an international level, advanced practice nurses, whose competencies extend beyond those of generalist nurses, address a wide range of healthcare challenges.^2^ To do so, advanced practice nurses acquire expertise, complex skills and advanced clinical competencies through graduate education (master’s degree or higher). Their roles, characteristics and scope are influenced by contextual factors within each country or setting.^2^

Monitoring the development of advanced practice nurses’ roles is an essential component of their implementation. By assessing their utilisation and impact, risks can be mitigated and the long-term sustainability of the role can be ensured.^3^ In countries such as Australia, Canada and the USA, advanced practice nurses are well established, federally regulated and formally recognised.^4^ In other countries, including Belgium, Cyprus, Germany, Spain and Austria, the development of these roles is still evolving.^5^ In countries in the early stages of development, there are few advanced practice nurses in active practice, limited robust data and continuing debate about the necessity of the role. Furthermore, the role is often inconsistently understood.^6^

In countries such as Germany and Spain, the implementation of advanced practice nurses remains fragmented and phenomenon-specific, often limited to individual hospitals, as legal frameworks are still being developed.^5^ Similarly, in Austria, advanced practice nursing is in its formative phase. No legal framework currently exists to expand the competencies of practicing advanced practice nurses, and the title remains unprotected and unregulated.^7^ Internationally, advanced practice nurse is considered a generic term encompassing distinct roles such as Nurse Practitioners and Clinical Nurse Specialists.^7^ However, in some countries, blended roles exist without clear delineation.^8^ This contributes to a heterogeneous understanding of advanced practice nursing roles, their definitions and operationalisation across Europe, influencing the competencies and responsibilities associated with these roles.^5^

In Switzerland, the first master’s programme for advanced practice nursing was introduced over 20 years ago. However, there remains no clear distinction between nurse practitioner and clinical nurse specialist roles, nor clarity regarding their scope of practice.^9^ To illustrate the current state of advanced practice nursing in Switzerland, Beckmann *et al*^9 10^ developed a survey to identify tasks performed by advanced practice nurses regardless of role. They also examined barriers and facilitators of advanced practice nursing and levels of job satisfaction. Findings showed that 37% of the participating advanced practice nurses frequently worked within the scope of a registered nurse. The lack of role clarity was identified as a major barrier to effective practice and a key factor influencing job satisfaction.^9^

Similar to Austria, in Germany the scope of practice for advanced practice nurses is limited, and the role is still evolving. Seismann-Petersen *et al*^11^ evaluated role clarity, competencies, autonomy and interprofessional collaboration among advanced practice nurses in Germany. The study included advanced practice nurses working in both primary and acute care settings and demonstrated that they have not yet reached internationally proposed standards. However, the perspectives of patients and healthcare providers were not included.^11^ Schwingrouber *et al*^12^ investigated activities, practice domains and the implementation processes of advanced practice nurses in France from the perspectives of advanced practice nurses, health professionals, leaders and decision-makers. This study emphasised the importance of systematic evaluation to support role development and implementation, and highlighted the need for comprehensive, system-wide strategies to enhance role clarity, autonomy and integration into clinical settings.

Evaluating the development of advanced practice nurses’ roles is a critical and ongoing process throughout implementation, as failure to evaluate may hinder both impact and sustainability.^3^ Brooten *et al*^6^ noted, evaluation outcomes should be selected carefully, as they may be influenced by dose effects such as the number of advanced practice nurses, patient contacts, education, acceptance and openness to change. In countries where role development and implementation are still in their infancy, detecting effects on patient outcomes or costs is often limited by small numbers of advanced practice nurses and regulatory constraints. Therefore, evaluations in these settings should focus on scope of practice, team impact and perceptions of the role from various stakeholders’ perspectives.^6^

Evaluations that focus on a specific role and are conducted within a defined clinical context may fail to capture a broader hospital system perspective that incorporates diverse stakeholder views and extends beyond individual roles or departments. For example, Dach *et al*^13^ evaluated the implementation of a nurse practitioner role within an inpatient surgical setting, primarily focusing on role-specific and setting-specific outcomes. While this mixed methods study provided valuable insights into an individual implementation context, it offers limited understanding of system-level perspectives and organisational change. To achieve a more comprehensive understanding of the multidimensional impacts of advanced practice nurses during the early stages of their implementation, a mixed methods study will be conducted to evaluate these roles and their implementation in an Austrian university hospital. This approach acknowledges the complexity inherent in advanced practice nurse roles and incorporates the perspectives of multiple healthcare providers, stakeholders and advanced practice nurses from various departments.

## Aims and research questions

The objective of this study is to evaluate the clinical, professional and organisational impact of implementing and developing the roles of advanced practice nurses on healthcare delivery in an Austrian university hospital. In this context, impact refers to the long-term and supra-individual effects^14^ of advanced practice nurse role development and implementation. The study will consider the perspectives of diverse healthcare professionals, stakeholders and advanced practice nurses from multiple departments, assessing their influence across clinical, professional and organisational dimensions.

This study is guided by the following research questions:

Over-reaching research question

- What are the perspectives of advanced practice nurses, nursing staff, nursing management and physicians on the clinical, professional and organisational impact of advanced practice nurses’ role development and implementation at the University Hospital Salzburg?

Quantitative research question

- What are the views of advanced practice nurses, nursing staff, nursing management and physicians on the clinical, professional and organisational impact of the development and implementation of advanced practice nurses’ roles at the University Hospital Salzburg?

Qualitative research question

- How do advanced practice nurses, nursing staff, nursing management and physicians describe the clinical, professional and organisational impact of advanced practice nurses’ role development and implementation at the University Hospital Salzburg?

Mixed methods research question

- In what ways do qualitative findings from interviews and focus groups help explain and expand understanding of the quantitative results regarding the clinical, professional and organisational impact of advanced practice nurses’ role development and implementation at the University Hospital Salzburg?

## Methods and analysis

**Figure 1:**
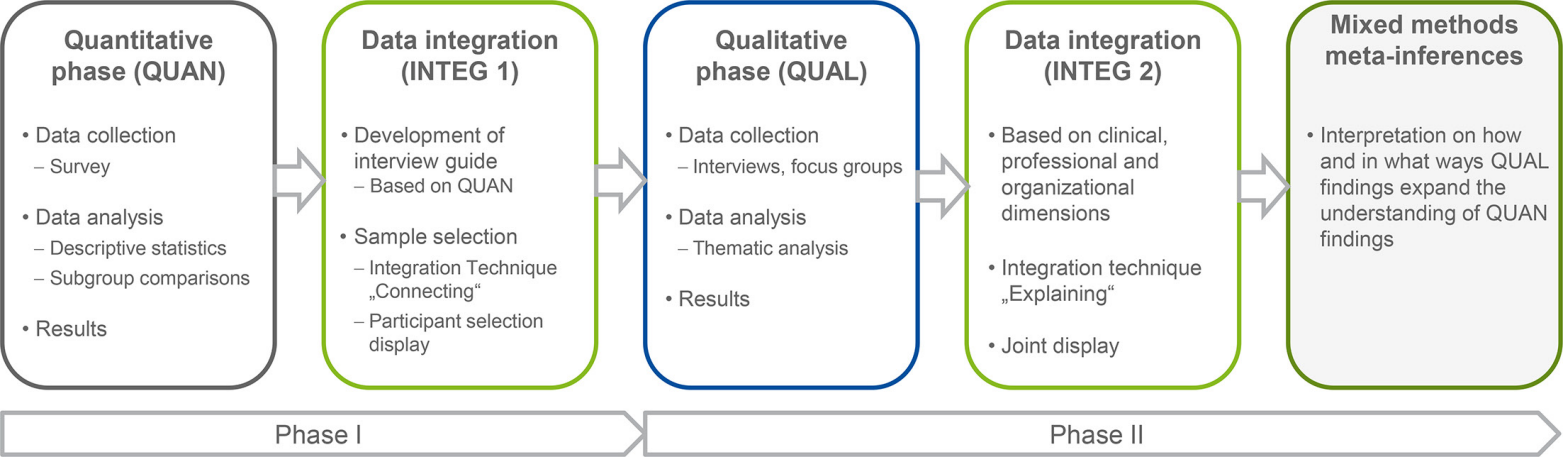
Explanatory sequential design process.

This mixed methods study will employ an explanatory sequential design grounded in the philosophical paradigm of pragmatism.^15^ In the initial quantitative phase (QUAN), survey data will be collected and analysed. The quantitative results will guide sampling procedures for the subsequent qualitative phase (QUAL) through the integration technique ‘Connecting’.^16 17^ Quantitative findings will also inform data collection strategies and analytical approaches in the qualitative phase.^18^ This represents the first point of integration (INTEG 1). The second integration point (INTEG 2) involves developing a joint display^17^ that integrates the clinical, professional and organisational dimensions. The explanatory sequential design process of the planned study is illustrated in figure 1.

### Framework

**Figure 2:**
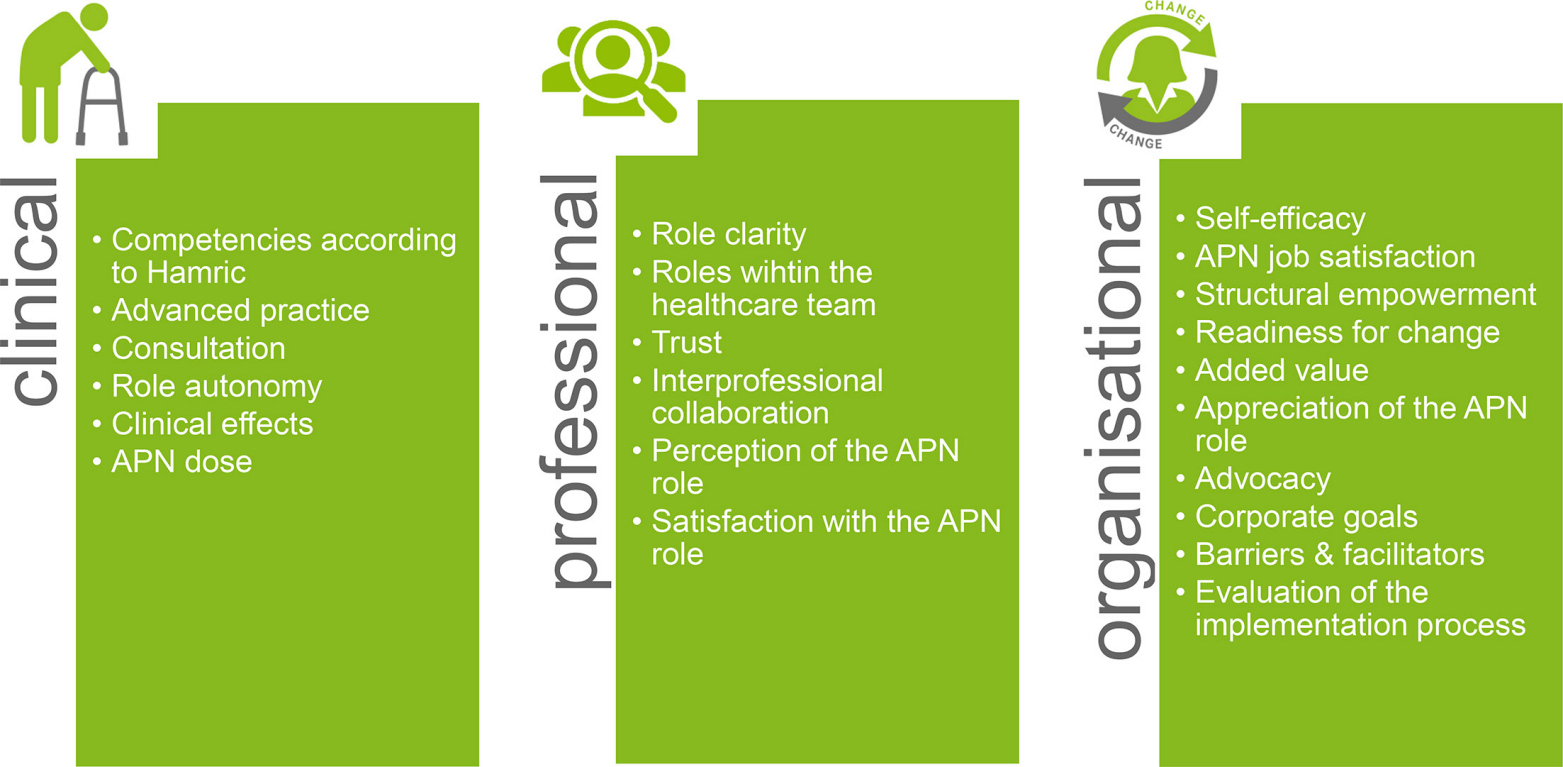
Conceptual framework. APN, advanced practice nurse.

To address the complexity of evaluating advanced practice nurses’ roles and role implementation, the authors developed a conceptual framework based on the ‘Participatory Evidence-Informed Patient-Centred Process for Advanced Practice Nurses Role Development–plus’^3^ and the framework for evaluating the impact of nurse consultant roles.^19^ The framework by Bryant-Lukosius *et al*^3^ was created to evaluate Swiss advanced practice nurses’ roles and incorporates evaluation aims, questions and tools that consider various roles and stages of development. The framework by Gerrish *et al*^19^ evaluates the impact of nurse consultant roles on clinical, professional and organisational outcomes, addressing both direct and indirect effects of advanced practice nurses. The conceptual framework for this study evaluates the impact of role development and implementation across clinical, professional and organisational dimensions. Within these dimensions, subdimensions identified from the literature form the foundation for quantitative and qualitative data collection, analysis and integration. Both the validated instruments and study-specific data collection tools are aligned with the subdimensions incorporated into the conceptual framework (figure 2).

### Quantitative phase (QUAN)

In the quantitative phase, an online survey will be administered to eligible participants.

#### Settings

In response to educational reforms introduced in Austria in 2016, registered nurse education was integrated into the tertiary education system. Consequently, registered nurses now study at universities of applied sciences and earn a bachelor’s degree.^20^ This led to the reorganisation of the skill and grade mix at the University Hospital Salzburg, which included integrating nurse assistants and developing academic nursing roles at both bachelor’s and master’s levels within direct clinical care. Since 2017, advanced practice nurses have been strategically implemented across designated departments of the University Hospital Salzburg. Role development and implementation have been guided by the ‘Participatory Evidence-Informed Patient-Centred Process for Advanced Practice Nurse Role Development Framework’^21^ and the central criteria and competencies outlined by Hamric.^22^ The study will be conducted across surgical, medical, interdisciplinary, psychiatric, paediatric and critical care departments of the University Hospital Salzburg.

#### Participants and recruitment

**Figure 3:**
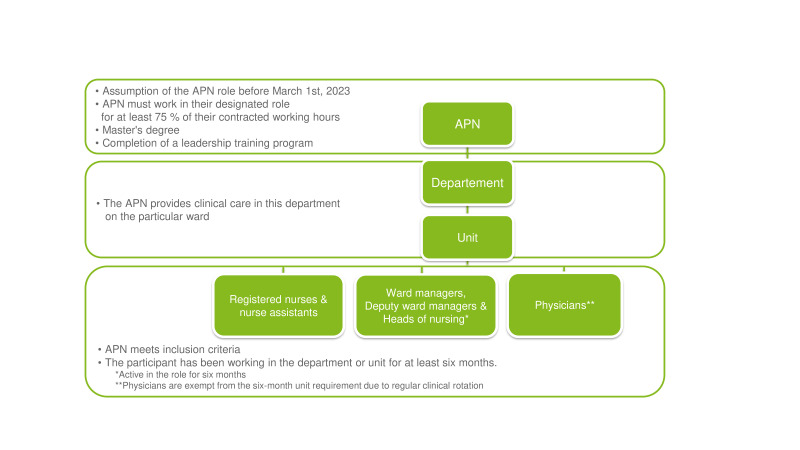
Inclusion criteria. APN, advanced practice nurse.

At the University Hospital Salzburg, a total of 22 advanced practice nurses are currently employed. Based on the organisational structure and staffing levels of the participating departments, the eligible population for the remaining cohorts comprises 498 registered nurses and nurse assistants, 49 nurse managers and 183 physicians. Participant recruitment will follow a top-down approach, beginning with the advanced practice nurses. If an advanced practice nurse in a department meets the inclusion criteria, all registered nurses, nurse assistants, nurse managers and physicians working in the respective advanced practice nurse’s clinical unit will also be eligible, provided they meet the inclusion criteria shown in figure 3. The study will employ a census, non-probabilistic design that includes all participants who meet the predefined selection criteria.^14^

#### Study instruments

Following a literature review, four validated instruments were selected based on relevance and two additional instruments were developed to address study-specific requirements. Table 1 offers an overview of the instruments employed in this study and their psychometric properties. The following section outlines the concepts and rationale for each validated instrument and describes the surveys specifically developed for this study.

**Table 1:** Quantitative data collection instruments

Instrument	Dimension (subdimension)	Cohort	Description	Psychometric properties	Analysis	Language	Items	Citation
APN-Task Questionnaire	Clinical (tasks according to Hamric)	APNs	APN activities based on Hamric’s framework Job satisfaction Barriers and facilitators of role implementation	Cronbach’s alpha 0.795–0.879	Frequencies and percentages for each item	EN	42	^10^
Occupational Self-Efficacy Scale (BSW-5-Rev)	Organisational (self-efficacy)	APNs	Confidence in fulfilling professional demands through personal competence	Cronbach‘s alpha 0.73–0.77	Calculation of unweighted mean scores	DE	5	^29^
Conditions for Work Effectiveness Questionnaire-Short Form	Organisational (structural empowerment)	APNs	Structural empowerment	Cronbach’s alpha 0.89	Calculation of mean scores	EN	21	^28^
Organisational Readiness for Implementing Change	Organisational (readiness for change)	APNs, registered nurses, nurse assistants, nurse managers, physicians	The organisational readiness for change is assessed by evaluating commitment to and confidence in successfully implementing change	Cronbach’s alpha 0.93	Calculation of sum scores	DE	10	^25^
Core (CARI) and Supplementary (SARI) Questionnaire on APN Role Development and Implementation	Clinical, professional, organisational	APNs, registered nurses, nurse assistants, nurse managers, physicians	Evaluates APN role-development and –implementation across clinical, professional and organisational dimensions	Survey development according to Moosbrugger and Kelava,^33^ face validity	Frequencies and percentages for each item; calculation of mean scores for each dimension	DE	CARI: 48	Self-developed
Clinical activities	Clinical (advanced practice)	APNs, nurse managers, physicians	Lists clinical activities that may be performed by APNs	Survey development according to Moosbrugger and Kelava,^33^ face validity	Frequencies and percentages for each item	DE	33	Self-developed

APN, advanced practice nurse; BSW-5-Rev, Revised German Scale for Measuring Occupational Self-Efficacy; CARI, Core Questionnaire on APN Role Development and Implementation; DE, German; EN, English; SARI, Supplementary Questionnaire on APN Role Development and Implementation.

### Organisational Readiness for Implementing Change

Organisational readiness for change is critical to the successful implementation of interventions, policies or programmes.^23^ As defined by Weiner *et al*,^23^ organisational readiness encompasses both the motivation and the perceived capability of organisational members to implement change. They emphasise that assessing only motivation is insufficient; the perceived capability—or efficacy—of members to enact change must also be considered.^23^ Organisational readiness for change is a key factor in the development and implementation of advanced practice nursing roles, as the institutional environment can either facilitate or hinder this process.^12^ Based on Weiner’s theory, the Organisational Readiness for Implementing Change instrument was developed to measure change commitment and change efficacy.^24^ The German version, translated and psychometrically tested by Lindig *et al*,^25^ will be administered to all participant cohorts to capture a comprehensive view of organisational readiness for implementing advanced practice nursing roles.

### Conditions for Work Effectiveness Questionnaire II

According to Kanter,^26^ work environments that provide access to information, resources, support and opportunities for growth are considered empowering. In such environments, employees are encouraged to apply their expertise and judgement, enhancing their ability to perform effectively and successfully.^27^ The Conditions for Work Effectiveness Questionnaire II measures structural empowerment based on Kanter’s theory. It evaluates employees’ perceived access to key organisational structures, including opportunity, information, resources and support, as well as formal and informal power. Empowerment is crucial for implementing advanced practice nurse roles, as managerial support and stakeholder engagement are key to successful integration.^12^ In this study, the Conditions for Work Effectiveness Questionnaire II measures how empowered advanced practice nurses feel in their work environment, focusing on access to resources, support, information and development opportunities.^28^

### Occupational Self-Efficacy Scale

The concept of self-efficacy, described by Bandura,^29^ refers to an individual’s belief in their capacity to perform actions necessary to achieve specific goals, particularly in challenging or demanding situations. Context-specific occupational self-efficacy reflects the individual’s confidence in meeting professional demands by relying on their own competencies.^30^ The occupational self-efficacy of advanced practice nurses will be assessed using Knispel’s^29^ Revised German Scale for Measuring Occupational Self-Efficacy (BSW-5-Rev).

### Advanced practice nurses: task questionnaire

Beckmann *et al*^10^ developed a questionnaire to map the tasks of advanced practice nurses based on Hamric’s core competencies^22^ and to evaluate job satisfaction and factors influencing role performance.^9^ The questionnaire comprises seven competency domains: (1) direct clinical practice, (2) guidance and coaching, (3) consultation, (4) collaboration, (5) leadership, (6) evidence-based practice and (7) ethical decision-making. It measures the frequency of advanced practice nurses’ activities within these domains over the past 30 days using a 4-point scale (never, occasionally, frequently, constantly).

Participants also estimate the proportion of their daily clinical practice spent in each competency area, ensuring the total equals 100%. Preparedness to apply each competency is assessed on a 7-point Likert scale (1=not prepared to 7=very well prepared). Job satisfaction is measured using a 4-point scale (very satisfied to very dissatisfied), and participants identify key factors affecting their satisfaction. Finally, they are invited to list up to three perceived barriers and facilitators to their role performance.^9^

As described by Beckmann *et al*,^10^ this questionnaire can be used independently of specific advanced practice nurse roles and is suitable for diverse healthcare settings. It enables the identification of activities performed and their frequency within each competency domain, providing insight into which competencies are most frequently applied and the extent to which advanced practice nurses engage in advanced nursing practice as defined by Hamric’s model. By revealing areas where advanced practice nurses are already engaged in advanced practice, this instrument highlights potential gaps and opportunities for further role development, professional support and organisational integration.

### Core questionnaire on advanced practice nurses role development and implementation

Because no standardised and validated German instrument existed to assess advanced practice nurses’ roles across clinical, professional and organisational dimensions from the perspectives of multiple stakeholders, the authors developed a tailored, literature-based survey to address this gap and ensure broad applicability across specialties and contexts. The instrument captures the complexity of advanced practice nurses’ roles and can be applied not only to advanced practice nurses but also to other healthcare providers. It builds on prior research and literature concerning the evaluation of advanced practice nurses’ roles.^31 32^ The questionnaire consists of 48 items covering clinical, professional and organisational dimensions of advanced practice nurses’ role development and implementation, in line with the conceptual framework of this study. Items are rated on a 5-point Likert scale ranging from 1 (‘disagree’) to 5 (‘agree’) with an additional ‘don’t know’ option. Example topics include interprofessional collaboration, leadership, contribution to organisational goals and perceived impact on patient care. Instrument development followed the framework of Moosbrugger and Kelava.^33^ Based on a comprehensive literature review, an initial pool of items was generated. To ensure clarity and eliminate potentially irrelevant items, the pool underwent cognitive pretesting using the think-aloud method with three individuals possessing scientific expertise. Subsequently, the survey instruments were revised through a consensus panel with project members from the partner university, UMIT TIROL. Preliminary empirical testing of the questionnaire was conducted with a small sample from the target population (n=16). The pretest within the ‘Advanced Practice Nurses’ Evaluation’ project aimed to assess the data collection process under real-world conditions, identify issues related to questionnaire design and response behaviour and gather participant feedback. The usability and functionality of the online survey tool were also evaluated.^14^ This process resulted in minor wording adjustments.

### Clinical activities

The Clinical Activities Questionnaire, developed by the research team, includes 33 clinical activities that may be performed by advanced practice nurses. Examples include prescribing analgesics, adjusting medication dosages and making decisions regarding patient admission or discharge. Participants indicate, on a 5-point Likert scale (1=disagree to 5=agree), the extent to which they consider these activities appropriate for advanced practice nurses. Because of Austria’s restrictive legal framework,^34^ insights from clinical practice are especially valuable for advocating an expanded scope of practice for advanced practice nurses. The questionnaire was developed following the same procedures as the Core Questionnaire, including expert review, cognitive pretesting using the think-aloud method and empirical testing with a target population sample.^14^

### Data collection

Healthcare professionals from relevant departments and units will be invited to participate in the online survey. Recruitment will occur through posters and flyers. Participants will receive an email containing the survey link and informed consent. Data collection will occur over a minimum of 8 weeks, with regular reminders sent throughout the period.

### Data analysis

Data analysis will be conducted using IBM SPSS Statistics V.29.0.2.0. Statistical analyses of validated instruments will follow the recommendations of the original authors. The core and supplementary questionnaires developed for this study will first be analysed at the item level to determine frequencies and percentages. Mean scores will then be calculated for the clinical, professional and organisational dimensions. Subgroup comparisons between dependent and independent variables will test for statistically significant differences based on scale levels and distribution characteristics. Depending on the level of measurement and data normality, χ^2^ tests, Mann-Whitney U tests, Kruskal-Wallis tests (with Bonferroni-Holm correction), or independent samples t-tests will be used. Normality of metric variables will be assessed using the Shapiro-Wilk test, which demonstrates high sensitivity, particularly in smaller samples.^35^ Statistical significance will be set at alpha (α) ≤0.05.

### Data integration on the first integration point (INTEG 1)

After completing the quantitative phase, the first integration point will involve identifying quantitative findings that require further clarification. Particular attention will be given to surprising, inconsistent or unexpected results, which will inform the design of the qualitative phase.^15^ In line with the explanatory sequential design, decisions regarding sample composition and the thematic focus of the interviews (dimensions) will be based on the quantitative results.^18^ Following the ‘Connecting’ integration technique, the quantitative data will be linked to the qualitative phase through purposive sampling.^36^ A participant selection display^16 17^ will be developed to identify quantitative findings that warrant deeper exploration and to guide the selection of participants for the qualitative phase.^15^ Quantitative findings will first be examined within each study population and subsequently across study populations to identify findings requiring qualitative follow-up and to determine which study populations should be included in the qualitative phase. Particular attention will be given to unexpected or contradictory findings, notably high or low mean scores, heterogeneous responses, descriptively noticeable differences between study populations and findings that deviate from existing literature. These findings will be integrated into a participant selection display in tabular form, documenting the quantitative results selected for follow-up, the rationale for their selection and the study populations from which participants will be recruited. The display will subsequently inform purposive sample selection and the thematic focus of the interview guides.

## Qualitative phase

To expand, explain and deepen the quantitative findings,^15^ the qualitative phase of this explanatory mixed methods design will explore healthcare professionals’ experiences concerning the clinical, professional and organisational effects of advanced practice nurses’ role development and implementation.

### Sampling/recruitment

All participant cohorts (advanced practice nurses, registered nurses, nurse assistants, nurse managers and physicians) included in the quantitative phase are considered potential sources for the qualitative phase. Consistent with the study’s objectives, the inclusion of advanced practice nurses in the qualitative phase is ensured. Based on the participant selection display,^16^ a purposive sampling approach will be employed, including only individuals who participated in the online survey and meet the inclusion criteria presented in figure 3.

### Data collection

Qualitative data collection is planned through semi-structured, face-to-face expert interviews^37^ with advanced practice nurses and focus group discussions^14 38^ with the other cohorts selected according to the quantitative findings. The specific formats may be refined based on the emerging quantitative results. Expert interviews aim to elicit advanced practice nurses’ experiential and practical knowledge, as well as their implicit understanding of institutional processes.^37^ Focus group discussions represent a structured form of group interview led by a moderator using a semi-structured interview guide and focused on a defined topic.^14^ These discussions aim to reveal participants’ personal and non-public opinions and attitudes^38^ and are particularly suitable for mixed-methods studies.^14^ Focus groups will ideally include between four and eight participants.

### Data analysis

From the current perspective, qualitative data analysis will be conducted using reflexive thematic analysis^39^ in MAXQDA (VERBI-Software). However, the approach may be refined depending on the quantitative results. Reflexive thematic analysis provides flexibility by allowing both inductive and deductive theme development, as well as latent and semantic coding, acknowledging that these approaches represent points along a continuum rather than opposing categories.^39^ A more deductive orientation to reflexive thematic analysis emphasises a researcher-informed or theory-informed perspective, in which the developed codes reflect conceptual ideas to be explored and interpreted within the data set. In such cases, frameworks or theories serve as an ‘*…*interpretative lens through which to code and make meaning of the data’ (38, p. 57). This approach was selected to enable a deeper exploration of the dimensions and subdimensions identified through the quantitative findings and to support a theory-informed interpretation of the qualitative data. Reflexive thematic analysis with a predominantly deductive orientation was chosen for its adaptability and capacity to capture complex and nuanced experiences related to implementing advanced practice nurses’ roles. Given the explanatory purpose of the qualitative phase and its integration with quantitative results, this method facilitates the identification of both semantic and latent themes, recognises the researcher’s active interpretive role^39^ and promotes understanding of the clinical, professional and organisational effects of advanced practice nurses’ role development and implementation.

### Data integration on the second integration point (INTEG 2)

Data integration at the second integration point (INTEG 2) aims to deepen and enhance quantitative findings through qualitative results. A joint display will be developed, incorporating the clinical, professional and organisational dimensions, to demonstrate the connection between quantitative and qualitative findings and to illustrate how qualitative insights enrich the understanding of quantitative results.^15^ The integration technique ‘explaining’ will be applied to gain deeper insights into quantitative data through qualitative findings, including mixed-methods meta-inferences.^16 17^

### Mixed methods validity

We will apply the mixed-methods quality criteria proposed by Creswell and Plano Clark^15^ to guide the methodological design and execution of this study. As both quantitative and qualitative processes are employed, phase-specific quality criteria will be observed. The quantitative phase will be guided by established quality criteria for quantitative research, including construct validity, internal validity, external validity and statistical inference validity, as described by Döring.^14^ Accordingly, validated instruments with established psychometric properties were included in this study. Limitations related to the use of self-developed instruments are addressed in the ‘Strengths and limitations’ section. The qualitative phase will be guided by the trustworthiness criteria of credibility, transferability, dependability and confirmability.^14^

## Ethics and dissemination

The study was reviewed by the Ethics Committee of the Federal State of Salzburg. The Ethics Committee classified the study as not requiring formal ethical approval or further review (415-EALL/4/233/3-2025). This decision was based on the fact that the study exclusively involves healthcare professionals, does not include patient participation or patient-related data and does not constitute a clinical trial. The study adheres to the ethical principles of the Declaration of Helsinki,^40^ and complies with the European Union General Data Protection Regulation.^41^ For both the qualitative and quantitative phases, informed consent will be obtained from all participants. They will receive detailed information regarding the study’s objectives, potential implications, voluntary participation and data confidentiality. Quantitative data will be anonymised, and qualitative data will undergo pseudonymisation before analysis. Study findings will be disseminated through publications in peer-reviewed scientific journals and presentations at national and international conferences. In addition, results will be shared with study participants through internal dissemination events organised within the institution.

## Discussion

This study protocol outlines an explanatory sequential mixed-methods study,^15^ designed to evaluate the clinical, professional and organisational impact of implementing and developing the roles of advanced practice nurses in selected departments of an Austrian university hospital. The study incorporates perspectives from advanced practice nurses and other healthcare professionals at the system level. During the quantitative phase, both validated and self-constructed survey instruments will be employed. The qualitative phase builds on the quantitative findings to provide deeper insight into the results.^15^ Qualitative data will be collected through semi-structured expert interviews and focus group discussions and analysed using reflexive thematic analysis.^39^ The integrated findings will be presented in a joint display.^15^

Given the ongoing development of advanced practice nurse roles in Austria, including the absence of clearly defined advanced competencies, scope of practice and regulatory structures,^7^ focusing solely on effectiveness outcomes may obscure the broader impact of advanced practice nurses and underestimate their potential contributions.^6^ Therefore, consistent with international recommendations,^6^ this study examines healthcare professionals’ experiences and the indirect effects of advanced practice nurses’ roles, including their influence on interprofessional collaboration, communication, contribution to organisational goals and the acceptance of advanced practice nurse roles.

From a methodological perspective, the development, implementation and evaluation of advanced practice nurse roles align with the characteristics of complex interventions. Complex interventions encompass multiple components, settings, stakeholder groups and levels of influence, with strong interdependencies between the intervention and its context.^42^ Skivington *et al*^42^ recommend moving beyond an exclusive focus on outcomes toward a more comprehensive approach that evaluates overall impact. Such an approach considers how the intervention functions, interacts with its context and contributes to system-level change, including shifts in relationships, policies or social norms.^42^ To address the organisational context, this study employs the Organisational Readiness for Implementing Change instrument to assess healthcare professionals’ readiness to implement advanced practice nurse roles in selected departments of the University Hospital Salzburg.^25^ Furthermore, structural empowerment will be assessed using the Conditions of Work Effectiveness Questionnaire II^28^ to determine how supportive advanced practice nurses perceive their organisational environment in relation to role implementation. To the best of our knowledge, this is the first study to apply both instruments specifically to evaluate advanced practice nurse roles and their impact within healthcare settings.

As the implementation of advanced practice nurses at the University Hospital Salzburg is ongoing, with new advanced practice nurses continually developing and establishing their roles, the results of this study may identify opportunities for optimisation and inform future implementation efforts. Insights regarding facilitators, barriers and areas for improvement may strengthen the evidence base and serve as a resource for other healthcare institutions seeking to introduce or enhance advanced practice nursing roles. This mixed-methods study provides an opportunity to deepen understanding of the impact of advanced practice nurses’ role development and implementation across multiple dimensions and from the perspectives of diverse healthcare professionals adopting a hospital-wide, system-level approach. This perspective is essential because the effectiveness, acceptance and sustainability of advanced practice nurse roles are influenced by contextual and organisational factors.^2^ Integrating quantitative and qualitative findings will enable a comprehensive, system-level understanding of role implementation within the hospital context. A purely quantitative design without a qualitative component is often insufficient for investigating complex interventions, as qualitative or mixed-methods approaches are necessary to explore questions extending beyond effectiveness.^42^ Accordingly, integrating quantitative and qualitative data will yield deeper insights into mechanisms of change, contextual dynamics and system-level effects.

### Strengths and limitations of this study

Creswell and Plano Clark^15^ identify several validity threats specific to explanatory mixed-methods designs, including the failure to recognise quantitative findings that require further explanation, to address contradictory or unexpected results and to establish a strong link between quantitative and qualitative phases. To mitigate these risks, the research team includes experts in nursing science and psychology with methodological expertise in qualitative, quantitative and mixed-methods research, as well as in advanced practice nurse role development. This interdisciplinary approach facilitates the consideration of a wide range of potential explanations when integrating and interpreting findings.

Despite these strengths, several contextual and methodological limitations must be acknowledged. The Austrian context is distinctive due to recent reforms in nursing education, which may restrict the transferability of results to other healthcare systems where advanced practice nursing roles are already well established. Additionally, because validated German versions were unavailable, the Advanced Practice Nurse Task Questionnaire^10^ and the Conditions of Work Effectiveness Questionnaire^28^ were administered in English to participating advanced practice nurses, which may pose comprehension challenges. However, the Advanced Practice Nurse Task Questionnaire has been successfully applied in Switzerland,^9^ and given the participants’ master’s-level education, sufficient English proficiency can be reasonably assumed. Furthermore, the study includes a self-developed questionnaire. Although it was designed collaboratively with experts and underwent multiple rounds of pretesting to ensure face validity, its psychometric properties have not yet been formally evaluated. Psychometric testing of the self-developed instruments is planned following completion of the quantitative data collection. This represents an additional methodological limitation that should be considered when interpreting the findings.
