# Supplementary material for: Evaluation of the role development and implementation of advanced practice nurses in an Austrian university hospital: a mixed methods research protocol
Source: BMJ Open. 2026 Jul 31;16(7):e119182. doi: 10.1136/bmjopen-2026-119182 (PMC13436101; doi:10.1136/bmjopen-2026-119182)
Supplement: Supplementary file 1 [file bmjopen-2026-119182.reviewer_comments.pdf]

PEER REVIEW HISTORY

BMJ Open publishes all reviews undertaken for accepted manuscripts. Reviewers are asked to complete a checklist review form and are provided with free text boxes to elaborate on their assessment. These free text comments are reproduced below.

ARTICLE DETAILS

Title (Provisional)

Evaluation of the role development and implementation of Advanced Practice Nurses in a Austrian university hospital: a mixed methods research protocol

Authors

Pann, Kathrin Julia; Gruber, Simone Elisabeth Elisabeth; Eßl-Maurer, Roland; Moser, Franziska; Ewers, Andre

---

VERSION 1 - REVIEW

**Reviewer**	**1**
**Name**	**Grech, Joseph**
**Affiliation**	**University of Malta, Department of Nursing**
**Date**	**01-May-2026**
**COI**	**None**

Thank you for the opportunity to read and review this study protocol which reports on the mixed-methods evaluation of the role development and implementation of Advanced Practice Nurses in a university hospital in Austria. This is well-written and detailed study protocol which adopts an appropriate study design. Please find below some comments and suggestions:

The title clearly denotes that the study is about the evaluation of the development and implementation of advanced practice nurses, however, at times the emphasis is solely on the evaluation of the implementation, e.g. in the abstract, pg.2, line 26.

Pg. 6, lines 6/7 to 9/10: This sentence needs further elaboration. This will also help to improve the link between this sentence and the next one.

Pg. 6, line 22/23: Given that the design has not been explained yet, I would suggest simply referring to it as ‘study’.

Some of the Research Questions can perhaps be made more concise, improving readability. For example, the overall RQ can be rephrased to “What are the perspectives of advanced practice nurses, nursing staff, nursing management, and physicians of the impact of the development and implementation of advanced practice nurses’ roles at the University

Hospital Salzburg on the clinical, professional, and organisational dimensions?" This is just a quick example. The Quantitative RQ seems to imply that the authors are interested in learning on how these stakeholders assess the impact of this role, rather on what are their views.

Participants and Recruitment – It would be helpful to know how many advanced practice nurses are employed at the hospital and the number of health care professionals (nurses and physicians) who may be eligible, if possible.

Study instruments – It would also be helpful for the reader if the tools used are clearly linked to the dimensions (and possibly the sub-dimensions) outlined in the conceptual framework of this study. Unlike the "Clinical Activities" Questionnaire, the "Core questionnaire on advanced practice nurses role development and implementation" lacks information on examples and scoring. There is also no information about the number of items in the text.

It would be helpful to have more information on the "Connection" integration technique.

Pg. 17, lines 36/37 to 39/40: This sentence is not clear on how it applies to the study. For example, I understood that the self-developed tools will not be psychometrically tested.

VERSION 1 - AUTHOR RESPONSE

Reviewer # 1		
**Comments**	Page	Response
**The title clearly denotes that the study is about the evaluation of the development and implementation of advanced practice nurses, however, at times the emphasis is solely on the evaluation of the implementation, e.g. in the abstract, pg.2, line 26.**	p.1	Thank you for highlighting this inconsistency. We revised the wording throughout the manuscript to ensure consistent reference to both role development and implementation. However, terminology derived from the cited literature was retained where sources specifically referred only to implementation and did not explicitly address role development.
**Pg. 6, lines 6/7 to 9/10: This sentence needs further elaboration. This will also help to improve the link between this sentence and the next one.**	p. 5	Thank you for bringing this to our attention. To better contextualise this statement, we provided additional details regarding the study by Dach et al.
**Pg. 6, line 22/23: Given that the design has not been explained yet, I would suggest simply referring to it as 'study'.**	p. 5	Thank you for your remark, we deleted the study design in the section "aims and research questions", and as suggested referred to it as "the study".
**Some of the Research Questions can perhaps be made more concise, improving readability. For example, the overall RQ**	p. 5 & 6	Thank you for bringing this to our attention. We rephrased the overall research question based on your suggestion and revised the

**can be rephrased to "What are the perspectives of advanced practice nurses, nursing staff, nursing management, and physicians of the impact of the development and implementation of advanced practice nurses' roles at the University Hospital Salzburg on the clinical, professional, and organisational dimensions?" This is just a quick example. The Quantitative RQ seems to imply that the authors are interested in learning on how these stakeholders assess the impact of this role, rather on what are their views.**		remaining research questions to improve readability.
**Participants and Recruitment – It would be helpful to know how many advanced practice nurses are employed at the hospital and the number of health care professionals (nurses and physicians) who may be eligible, if possible.**	p. 8	We appreciate this comment on the "Participants and Recruitment" section. To improve transparency concerning the study population, we added the following information to the manuscript:<br><br>At the University Hospital Salzburg, a total of 22 advanced practice nurses are currently employed. Based on the organisational structure and staffing levels of the participating departments, the eligible population for the remaining cohorts comprises 498 registered nurses and nurse assistants, 49 nurse managers, and 183 physicians.
**Study instruments – It would also be helpful for the reader if the tools used are clearly linked to the dimensions (and possibly the sub-dimensions) outlined in the conceptual framework of this study. Unlike the "Clinical Activities" Questionnaire, the "Core questionnaire on advanced practice nurses role development and implementation" lacks information on examples and scoring. There is also no information about the number of items in the text.**	p. 9 - 11<br><br><br><br><br>p.13 & 14	Thank you for your relevant comment. In Table 1, the instruments were linked to the corresponding dimensions and, where possible, to the respective subdimensions of the conceptual framework.<br><br>As suggested, we added information regarding the number of items, scoring, and examples of assessed topics to the section "Core questionnaire on advanced practice nurses role development and implementation".
**It would be helpful to have more information on the "Connection" integration technique.**	P. 15	We agree that this aspect requires further explanation. We added a definition of the connecting technique. To clarify our analytical approach to the technique

		"connecting" at the first integration point, we further specified how quantitative findings will be examined within and across study populations and which findings will be considered surprising, heterogeneous or requiring further explanation. These findings will guide both participant selection and the thematic focus of the interview guides.
**Pg. 17, lines 36/37 to 39/40: This sentence is not clear on how it applies to the study. For example, I understood that the self-developed tools will not be psychometrically tested.**	p. 17, 18	Thank you for this important comment. We revised the wording to clarify that the quantitative phase is guided by established quality criteria for quantitative research rather than implying full psychometric validation of all instruments. The limitations related to the self-developed instruments are additionally addressed in the strengths and limitations section. We added a reference to the limitation section. We hope that you will find these adaptions sufficient.<br><br>We understand that the previous wording in the section strengths and limitations may have suggested that the self-developed instruments would not undergo psychometric evaluation at all. To clarify this point, we revised the manuscript to explicitly state that psychometric testing of the developed instruments is planned following completion of the quantitative data collection. This pragmatic approach nevertheless represents a methodological limitation of the study.

VERSION 2 - REVIEW

Reviewer 1

Name Grech, Joseph

Affiliation University of Malta, Department of Nursing

Date 15-Jul-2026

COI

Thank you for the opportunity to read and review this manuscript again. I am also glad that the authors have found most of my suggestions and comments useful. My concerns have been adequately addressed. Kindly note that the quantitative research question is practically the same as the overarching research question.
